# Supplementary material for: The Fractional exhaled Nitric Oxide (FeNO)- test as add-on test in the diagnostic work-up of asthma: a study protocol
Source: BMC Pulm Med. 2024 Apr 15;24:178. doi: 10.1186/s12890-024-02990-2 (PMC11020987; doi:10.1186/s12890-024-02990-2)
Supplement: Supplementary file 2 — Supplementary Material 2 [file 12890_2024_2990_MOESM2_ESM.docx]

**The Fractional Exhaled Nitric Oxide (FeNO)- test as add-on test in the diagnostic work-up of asthma:**

A study protocol

**T. (Tuba) Kaya^1^, G.J. (Gert-Jan) Braunstahl^1,2^, J.C.C.M. (Johannes) in ’t Veen^1,2^, J.H. (Jasper) Kappen^1,3^, J.P.M. (Hanna) van der Valk^1,2*^**

1. *Department of Pulmonary Diseases, Center of Excellence for asthma, COPD and respiratory allergy, Franciscus Gasthuis & Vlietland, Rotterdam, The Netherlands*
2. *Department of Pulmonary Diseases, Erasmus Medical Centrum, Rotterdam, The Netherlands*
3. *National Heart and Lung Institute, Imperial College London; Asthma UK Centre in Allergic Mechanisms of Asthma*

*J.P.M. van der Valk

Franciscus Gasthuis & Vlietland

Department Pulmonary diseases

P.O. box 3045 PM Rotterdam

The Netherlands

Phone +31642912700

E-mail: h.kuiper-vandervalk@franciscus.nl

**Word count:**

2459

**Abstract (401 words)**

**Background**

Asthma is a common disease characterized by chronic inflammation of the lower airways, bronchial hyperactivity, and (reversible) airway obstruction. The Global Initiative of Asthma Guideline recommends a flowchart to diagnose asthma with first-step spirometry with reversibility and a bronchial challenge test (BPT) with histamine or methacholine as a second step [1]. The BPT is considered burdensome, time-consuming for patients and staff, can cause side effects, and is expensive. In addition, this test strongly encumbers lung function capacity. Elevated Nitric Oxide (NO) is associated with airway eosinophilic inflammation in asthma patients and can be measured in exhaled air with the Fractional exhaled (Fe) NO-test. This low-burden FeNO-test could be used as an ‘add-on’ test in asthma diagnostics [2, 3].

**Methods and analysis**

This multi-center prospective study (Trial number: NCT06230458) compares the ‘standard asthma diagnostic work-up’ (spirometry with reversibility and BPT) to the ‘new asthma diagnostics work-up’ (FeNO-test as an intermediate step between the spirometry with reversibility and the BPT), intending to determine the impact of the FeNO-based strategy, in terms of the number of avoided BPTs, cost-effectiveness and reduced burden to the patient and health care.

The cost reduction of incorporating the FeNO-test in the new diagnostic algorithm will be established by the number of theoretically avoided BPT. The decrease in burden will be studied by calculating differences in the Visual Analogue Scale (VAS) -score and Asthma Quality of Life Questionnaire (AQLQ) -score after the BPT and FeNO-test with an independent T-test. The accuracy of the FeNO-test will be calculated by comparing the FeNO-test outcomes to the (gold standard) BPTs outcomes in terms of sensitivity and specificity. The intention is to include 171 patients.

**Ethics and dissemination**

The local medical ethics committee approved the proposed study and is considered a low-burden and risk-low study. The local medical ethics committee registration number: R23.005.

**Strengths and limitations of this study**

Strengths

This is the first study that investigates the value of the FeNO-test (cut off ≥ 50 ppb) as an add-on test, to determine the impact of the FeNO-based strategy, in terms of the number of avoided BPTs, cost-effectiveness, and reduced burden on the patient and health care.

Limitations

High FeNO levels may also be observed in other diseases such as eosinophilic chronic bronchitis and allergic rhinitis. The FeNO-test can be used to rule in a diagnosis of asthma with confidence, however, due to the poor sensitivity it is not suitable to rule out asthma.

**Keywords**

Asthma, diagnostics, bronchial provocation test, Fractional exhaled Nitric Oxide-test, cost-effectiveness, burdensome

**Background**

Asthma is a common disease with an increase in prevalence worldwide [4]. Diagnosing asthma in time (early recognition) is relevant to prevent deterioration of the lungs and to be able to treat the patient adequately.

The GINA guideline recommends a flowchart to diagnose asthma (‘standard diagnostic work-up’) [1]. The first step in the diagnostic asthma work-up is to examine the detailed history of the patients. If the history supports asthma, spirometry with tested reversibility using a bronchodilator is recommended. Reversibility is demonstrated if the bronchodilatation test is positive (delta FEV1 ≥ 200 ml and ≥ 12%). The next step in the diagnostic procedure is the BPT to demonstrate bronchial hyperresponsiveness [1]. The BPT is considered positive if the FEV1 decreases by at least 20% following inhalation of ≤ 2.39 mg of histamine or ≤ 1.87 mg of methacholine. Approximately 60% of the patients with probable asthma has a positive BPT [5].

However, BPT is expensive, time-consuming, unpleasant for the patient, and can cause side effects [6]. In addition, most lung function departments in the Netherlands have limited capacity to perform all the needed BPTs resulting in delays in adequate diagnostic evaluation for the patient. Therefore, there is an urgent need for a new diagnostic asthma work-up to be implemented in asthma care.

Next to bronchial obstruction and hyperresponsiveness, bronchial wall inflammation also plays a role in asthma. Several inflammatory cells are involved in this process, such as lymphocytes, dendritic cells, mast cells, and eosinophils. Nitric oxide (NO) is associated with airway eosinophilic inflammation in asthma patients and can be measured in exhaled air with the FeNO-test [7].

The FeNO-test is currently not included in the diagnostic flowchart of the GINA guideline, as the BPT is still considered the gold standard [1]. Various studies show a sensitivity for FeNO between 85 and 88% and a specificity between 79 and 90% to diagnose asthma [2, 8]. In an appropriate clinical context and a FeNO above 50 ppb, the chance of a correct asthma diagnosis is 96% [8].

This study (Trial number: NCT06230458) investigates the value of the FeNO-test (cut off ≥50 ppb) as an add-on test, intending to determine the impact of the FeNO-based strategy, in terms of the number of avoided BPTs, cost-effectiveness, and reduced burden on the patient and healthcare. The FeNO-test can be used to rule in a diagnosis of asthma with confidence, however, the poor sensitivity does not allow asthma to be ruled out. Although the accuracy of the FeNO-test is extensively proven in previous studies, it is included for verification in this study.

The hypothesis is that the BPT will not be necessary for about 50% of the patients. This percentage will probably have a FeNO test result with a value of ≥ 50 ppb. These will be the patients with Type-2 high asthma (allergic/eosinophilic asthma). This type of asthma is the most common: about 50%-70% of people with asthma have an underlying type 2 inflammation [9].

The UK National Institute for Health and Care Excellence (NICE) asthma guidelines recommend the use of the FeNO-test in the diagnostic asthma work-up[10]. Also, the ERS task force recommends the measurement of FeNO as part of the diagnostic work-up of children aged 5–16 years and adults with suspected asthma[2, 3]

The ultimate goal of this study is to implement the FeNO-test also in the GINA- and National asthma guidelines if this study demonstrates that incorporating the FeNO-test in the diagnostic work-up for asthma has favourable effects in terms of the number of avoided BPTs, cost-effectiveness, and reduced burden on the patient and healthcare.

**Methods/Design**

Inclusion

Patients ≥ 18 years old with a probable history of asthma visiting the pulmonary outpatient ward/ clinic will be asked to participate in this study. Patients with a confirmed diagnosed asthma are not allowed to participate. The inclusion of patients with respiratory infections <3 weeks ago will be postponed to >3 weeks. For reasons of external validity and generalizability of the study results, it was decided not to exclude subgroups such as smokers or obese patients. Informed consent to participate will be obtained from the patients.

Study design

The study starts with a standard care visit in which all patient characteristics are collected. During this visit, blood will be collected to measure total IgE (tIgE), specific IgE (sIgE) to inhalation allergens, and eosinophils to map the asthma profile of the patient. After that, all patients undergo spirometry with reversibility. If there is no reversibility, the patient will ask to participate in the study.

In a separate appointment, the BPT test (standard care) and FeNO-test (extra) will be done, and the diagnostic burden will be measured (Visual Analogue Scale (VAS)) after each test. After the study visit, the patient will receive the results of the FeNO-test and the BPT and will be treated

if they are diagnosed with asthma or get extra investigations if there is no asthma (standard care 2). After 3 months, patients will be medically controlled by the pulmonary physician for evaluating treatments and quality of life with the Asthma Control Questionnaire (ACQ) and Asthma Quality of Life (AQLQ) (standard consult 3, digital consult), and 2 additional questionnaires will be administered. The study program and diagnostic results are shown in Table 1 and Figure 1.

**Table 1. Study program**

| Standard care 1. | - Consult pulmonary physician - Blood testing - Spirometry |
| --- | --- |
| **Study visit 1** | - BPT (standard care) - Informed consent - FeNO-test (extra) - Questionnaires (VAS) (2, extra) |
| Standard care 2. | - Results of the FeNO-test and BPT - Diagnosis - Possible treatment - Possible additional investigations |
| Standard care 3. | - ACQ, AQLQ after 3 months of treatment (digital consult) |

FeNO-tests

The FeNO-test (Niox-Vero, Aerocrine AB, Sweden) will be measured at a constant flow rate of 50 mL/s and expressed as ppb following the guidelines [11, 12]. The FENO measurements are performed with a deep inhalation through the mouth and slow exhalation (constant flow rate of 50 mL/s). The measurement will be repeated if the patient does not blow with a constant flow rate of 50 mL/s. The FeNO meter shows whether the correct flow has been achieved. It is not allowed to eat any nitrate-rich food, to drink alcohol 2 hours before the test and to smoke 4 hours before the test. The FeNO-test will be postponed if the patient has a cold on the day of the examination. These factors will affect the test results.

Bronchial provocation test

The BPT is used as the gold standard in asthma diagnostics [1]. The BPT demonstrates bronchial hyperresponsiveness and is considered positive if the FEV1 decreases by at least 20% following inhalation of ≤ 2.39 mg of histamine or ≤ 1.87 mg of methacholine [13, 14].

Outcomes

The reduction in the number of provocation tests will be calculated by adding up the number of theoretically saved provocation tests. The decrease in direct and indirect costs will be calculated by adding up the costs of the number of provocation tests and the absence days, which were not necessary afterward, minus the extra costs of the FeNO measurement. The reduction in patient burden will be assessed with questionnaires. Asthma control and quality of life will be measured in the different groups using questionnaires. The accuracy of the FeNO test will be calculated by comparing the FeNO test results with the gold standard results of the challenge test.

Sample size calculation

The sample size calculation is based on the prevalence, sensitivity, and specificity based on the percentages in the literature. A prevalence of asthma is taken at 0.39, the sensitivity of the FeNO-test at 0.6 (mean of 0.5- 0.7 (for patients with type-2 high asthma)), and the specificity at 0.85 (mean of 0.75 to 0.95). This leads to 32.55% avoidable BPTs and an associated sample size is 150 patients. When we take the margins into account and accept a CI of 0.15, this leads to percentages avoidable BPT of 16.9 – 49.2% and an associated sample size of 96 – 171 patients. We have the intention to include 171 patients. The sample size calculation with all margins is shown (Supplementary 1).

Statistical analysis

Patient- and study characteristics will be reported in percentages, means, and standard deviation. The cost reduction of incorporating the FeNO-test in the new diagnostic algorithm will be established by the number of theoretically avoided BPT, theoretically avoided days of absenteeism caused by the BPT, and extra FeNO measurements. The decrease in burden will be studied by calculating differences in VAS-score and AQLQ-score after the BPT and FeNO-test with an independent T-test. The mean delta ACQ after 3 months of treatment in the patients with asthma and without asthma will be determined and also compared with an independent T-test. The accuracy of the FeNO-test will be calculated by comparing the FeNO-test outcomes to the (gold standard) BPTs outcomes in terms of sensitivity and specificity. A p-value of 0.05 will be considered statistically significant. The post-hoc analysis will be performed for subgroups of the patients; smokers and obese patients.

Patient and public involvement

The Dutch Advisory Council of the patient representation was involved in the development of this study protocol and will be permanently involved during the study. A patient information session will be organized twice a year, in which this research project is evaluated. Patient Reported Experience Measures (PREMs) Patient Reported Outcome Measures (PROMs) will be used in this evaluation.

**Discussion**

This study is the first care evaluation study that aims to calculate the cost-reduction and reduction in burdensome for the patient if the FeNO-test is implemented in the diagnostic work-up for asthma.

The FeNO-test is not (not yet) included in the asthma diagnostic flowchart of the GINA guideline, because the FeNO-test cannot replace the BPT (gold standard) entirely [15].

However, the UK National Institute for Health and Care Excellence (NICE) asthma guidelines recommend the use of the FeNO-test in the diagnostic asthma work-up [10]. Also, the ERS task force recommends the measurement of FeNO as part of the diagnostic work-up of children aged 5–16 years and adults with suspected asthma[2, 3].

A FeNO-test cut-off value above 40-50 ppb yields a specificity between 0.75 to 0.95 and a sensitivity between 0.19 to 0.81 for a diagnosis of asthma [2]. The high variability observed across the studies reflected differences in patient inclusion criteria in demographics, such as smoking and atopy status, or concurrent inhaled corticosteroid treatment during assessment [16, 17]. High FeNO levels may also be observed in other diseases such as eosinophilic chronic bronchitis and allergic rhinitis. The FeNO-test can be used to rule in a diagnosis of asthma with confidence, however, the poor sensitivity does not allow asthma to be ruled out [15].

This is the first study that investigates the value of the FeNO-test (cut off ≥50 ppb) as add-on test, intending to determine the impact of the FeNO-based strategy, in terms of the number of avoided BPTs, cost-effectiveness, and reduced burden on the patient and health care.

The total economic burden of asthma is high due to the prevalence of the disease with direct costs e.g. emergency visits, physician visits, and diagnostic tests, and indirect costs e.g. work and school absence [18]. Therefore, it is important to reduce costs. Preventing BPTs by adding the FeNO-test as an extra diagnostic tool in de asthma diagnostic work-up will substantially decrease asthma costs for health care by speeding up the diagnostic process. Significant costs can be avoided by saving the challenge test in the expected 50% of the patients as they will have a FeNO- test result with a value of ≥ 50 ppb. These will be the patients with Type-2 high asthma (allergic/eosinophilic asthma). This type of asthma is the most common type of asthma: about 50%-70% of people with asthma have an underlying type 2 inflammation [9].

Moreover, the BPT is unpleasant for the patient and not without side effects. BPT can cause symptoms such as wheezing, pharyngeal itching, cough, or chest tightness with sometimes persistence of symptoms for several days. This is not the case with the FeNO-test and these side effects can therefore be avoided in a significant number of patients. Three studies in a meta-analysis of a Dutch Cochrane review showed no difference in the quality of life between the group with a FeNO-test and the control group (no diagnostic intervention) (MD=0.02; 95% CI -0.10 to 0.14), which demonstrates that the de FeNO-test is a low-burden test for the patient [6]. The FeNO-test is considered low-burden, fast, and ensures cost reduction compared to the BPT [6].

A limited capacity is also available in most lung function labs in the Netherlands for BPTs. Since lung function technicians are scarce in the Netherlands, patients face long waiting lists. This is very patient-unfriendly and can lead to temporary undertreatment of the patients.

The limitation of the new diagnostic approach is that a small group of patients is at risk of being misdiagnosed with a false-positive FeNO-test in the future if the BPT is no longer performed for the asthma diagnostic control as in this study context. The size of this group depends on the accuracy of the FeNO-test. The assumption is a specificity of 90% (10% false positive FeNO-test)[2]. This is a sketch based on the mean percentages from the literature. The patients will be checked whether they have an effect on the treatment after 3 months and if there are doubts about the treatment effect then to perform the BPT.

In conclusion, this is the first study that investigates the value of the FeNO-test (cut off ≥50 ppb) as an add-on test in asthma diagnostics. The hypothesis is that the BPT will not be necessary for about 50% of the patients. This will substantially reduce asthma costs and burden for the patient and healthcare professionals. The ultimate goal is to adjust the GINA - and National asthma guidelines with the new diagnostic work-up. The current asthma diagnostic work-up is unsustainable in the future, because of the increase in the number of asthma patients, increase in healthcare costs, and increasing lung function waiting lists.

**Ethics and dissemination**

The local medical ethics committee; Medical Research Ethics Committees United (MEC-U), approved the proposed study (registration number R23.005).

This study has a low burden for the participating patients because the new diagnostic work-up does not differ much from the standard diagnostic work-up. Only the FeNO-test (5 minutes), a very simple and low-burden lung function test is extra. The worst side effect of the FeNO-test is light-headedness. The blood sampling is the same as in routine diagnostics for asthma. The patient could get a hematoma and sometimes patients have a vasovagal collapse. Also, the BPT test (70 minutes) is routine diagnostics for asthma. Side effects are dyspnea symptoms/ hyperresponsivity. The patients' questionnaires will take 5 minutes after each diagnostic test and after 3 months. We expect no risk for the study patients.

**Declarations':**

Ethics approval and consent to participate:

The local medical ethics committee; Medical Research Ethics Committees United (MEC-U), approved the proposed study (registration number R23.005). Informed consent to participate will be obtained from the patients.

Consent for publication:

Not applicable.

Availability of data and materials:

The data supporting this study's findings are available from the corresponding author, [J.P.M. van der Valk], upon reasonable request.

Competing interests:

There is no conflict of interest.

Funding:

This study is sponsored by ‘Bevordering Onderzoek Franciscus’ (charitable).

Authors' contributions:

T. (Tuba) Kaya Substantial contributions to drafting the work.

G.J. (Gert-Jan) Braunstahl Substantial contributions to the study design, drafting the work, and reviewing it critically for important intellectual content.

J.C.C.M. (Hans) in ’t Veen Substantial contributions to the study design, reviewing the article critically for important intellectual content.

J.H. (Jasper) Kappen Substantial contributions to the study design, reviewing the article critically for important intellectual content.

J.P.M. (Hanna) van der Valk Substantial contributions to the study design, drafting the work, and reviewing it critically for important intellectual content.

**List of abbreviations**

Asthma Quality of Life Questionnaire AQLQ

Bronchial challenge test BPT

Fractional exhaled Nitric Oxide FeNO

Global Initiative of Asthma Guideline GINA

Nitric Oxide NO

Patient Reported Experience Measures PREMs

Patient Reported Outcome Measures PROMs

specific IgE sIgE

Total IgE tIgE

Visual Analogue Scale VAS

**References**

1. *<https://ginasthma.org/2023-gina-main-report/>.*

2. Louis, R., et al., *European Respiratory Society Guidelines for the Diagnosis of Asthma in Adults.* Eur Respir J, 2022.

3. Gaillard, E.A., et al., *European Respiratory Society clinical practice guidelines for the diagnosis of asthma in children aged 5-16 years.* Eur Respir J, 2021. **58**(5).

4. Braman, S.S., *The global burden of asthma.* Chest, 2006. **130**(1 Suppl): p. 4S-12S.

5. Bins, J.E., et al., *The use of a direct bronchial challenge test in primary care to diagnose asthma.* NPJ Prim Care Respir Med, 2020. **30**(1): p. 45.

6. Damen J, H.P., Spijker R, Jenniskens K, Kamm I, Nijman S, Vernooij R. Nut van FeNO bij de diagnostiek en behandeling van specifieke groepen astmapatiënten. Nederlands Cochrane. 2021;1-400.

7. Malinovschi, A., et al., *Exhaled nitric oxide levels and blood eosinophil counts independently associate with wheeze and asthma events in National Health and Nutrition Examination Survey subjects.* J Allergy Clin Immunol, 2013. **132**(4): p. 821-7 e1-5.

8. Travers, J., et al., *Reference ranges for exhaled nitric oxide derived from a random community survey of adults.* Am J Respir Crit Care Med, 2007. **176**(3): p. 238-42.

9. Canonica, G.W., et al., *Defining type 2 asthma and patients eligible for dupilumab in Italy: a biomarker-based analysis.* Clin Mol Allergy, 2021. **19**(1): p. 5.

10. in *2018 exceptional surveillance of asthma: diagnosis, monitoring and chronic asthma management (NICE guideline NG80)*. 2018: London.

11. American Thoracic, S. and S. European Respiratory, *ATS/ERS recommendations for standardized procedures for the online and offline measurement of exhaled lower respiratory nitric oxide and nasal nitric oxide, 2005.* Am J Respir Crit Care Med, 2005. **171**(8): p. 912-30.

12. Ragnoli, B., et al., *Fractional nitric oxide measurement in exhaled air (FeNO): perspectives in the management of respiratory diseases.* Ther Adv Chronic Dis, 2023. **14**: p. 20406223231190480.

13. Sterk, P.J., *Virus-induced airway hyperresponsiveness in man.* Eur Respir J, 1993. **6**(6): p. 894-902.

14. Coates, A.L., et al., *ERS technical standard on bronchial challenge testing: general considerations and performance of methacholine challenge tests.* Eur Respir J, 2017. **49**(5).

15. Karrasch, S., et al., *Accuracy of FENO for diagnosing asthma: a systematic review.* Thorax, 2017. **72**(2): p. 109-116.

16. Dweik, R.A., et al., *An official ATS clinical practice guideline: interpretation of exhaled nitric oxide levels (FENO) for clinical applications.* Am J Respir Crit Care Med, 2011. **184**(5): p. 602-15.

17. Cordeiro, D., et al., *Utility of nitric oxide for the diagnosis of asthma in an allergy clinic population.* Allergy Asthma Proc, 2011. **32**(2): p. 119-26.

18. Hakansson, K.E.J., et al., *Beyond direct costs: individual and societal financial burden of asthma in young adults in a Danish nationwide study.* BMJ Open Respir Res, 2023. **10**(1).
